# Initial Mechanisms for the Unimolecular Thermal Decomposition of 2,6-Diamino-3,5-dinitropyrazine-1-oxide

**DOI:** 10.3390/molecules24010125

**Published:** 2018-12-31

**Authors:** Nianshou Cheng, Qiang Gan, Qian Yu, Xuemei Zhang, Rong Li, Shichuan Qian, Changgen Feng

**Affiliations:** 1 State Key Laboratory of Explosion Science and Technology, Beijing Institute of Technology, Beijing 100081, China; gdxqsh2018@163.com (N.C.); lirong2016@sohu.com (R.L.); qianshch2016@126.com (S.Q.); 2 School of Chemical and Material Engineering, Anhui Science and Technology University, Fengyang 233100, Anhui, China; zhangxm_ahstu@sina.com; 3 Institute of Chemical Materials, China Academy of Engineering Physics, Mianyang 621900, Sichuan, China; ddls2005@163.com

**Keywords:** LLM-105, initial channels, thermal decomposition mechanism, quantum chemical calculations

## Abstract

The initial channels of thermal decomposition mechanism of 2,6-diamino-3,5-dinitropyrazine-1-oxide (LLM-105) molecule were investigated. The results of quantum chemical calculations revealed four candidates involved in the reaction pathway, including the C–NO_2_ bond homolysis, nitro–nitrite rearrangement followed by NO elimination, and H transfer from amino to acyl O and to nitro O with the subsequent OH or HONO elimination, respectively. In view of the further kinetic analysis and ab initio molecular dynamics simulations, the C–NO_2_ bond homolysis was suggested to be the dominant step that triggered the decomposition of LLM-105 at temperatures above 580 K. Below this temperature, two types of H transfer were considered as the primary reactions, which have advantages including lower barrier and high rate compared to the C–NO_2_ bond dissociation. It could be affirmed that these two types of H transfer are reversible processes, which could buffer against external thermal stimulation. Therefore, the excellent thermal stability of LLM-105, that is nearly identical to that of 1,3,5-triamino-2,4,6-trinitrobenzene, can be attributed to the reversibility of H transfers at relatively low temperatures. However, subsequent OH or HONO elimination reactions occur with difficulty because of their slow rates and extra energy barriers. Although nitro–nitrite rearrangement is theoretically feasible, its rate constant is too small to be observed. This study facilitates the understanding of the essence of thermal stability and detailed decomposition mechanism of LLM-105.

## 1. Introduction

Thermal decomposition mechanisms for energetic materials, and their corresponding series of intermediates and products, are extremely important because they guide systematic predictions about unknown explosives, influence feasibility of large-scale synthesis, predict long-term stability for purposes of storage, and predict sensitivity to various stimuli, such as heat and mechanical impact [1]. The safety and performances of explosives are intimately associated with the thermal decomposition processes; therefore, knowledge of mechanistic pathways can assist researchers to understand the behaviors of explosives rather than by empirical ways.

**Figure 1:**
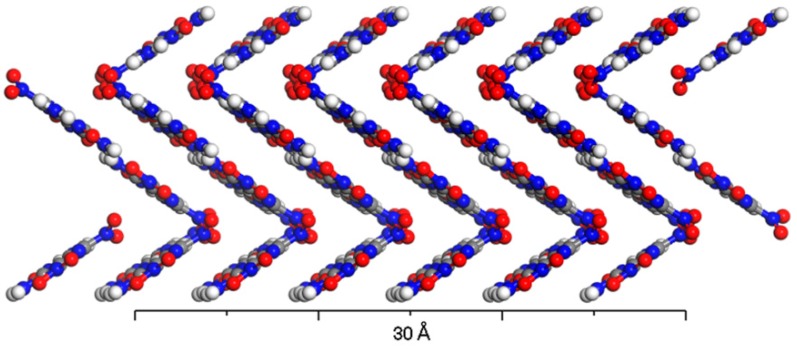
Crystal structure of LLM-105.

2,6-Diamino-3,5-dinitropyrazine-1-oxide (code: LLM-105), which was first synthesized by P.F. Pagoria [2] in 1995, is an explosive material with good performance. It was found to be a stable species with a high differential scanning calorimetry (DSC) decomposition temperature peak at 342 °C [3], equivalent to the stability of trinitrotoluene (TNT). Tarver et al [4] found that the thermal sensitivity of LLM-105 is between that of octahydro-1,3,5,7-tetranitro-1,3,5,7-tetrazine (HMX) and 1,3,5-triamino-2,4,6-trinitrobenzene (TATB). However, the impact sensitivity test showed that the Dh_50_ was up to 117 ± 1.0 cm, indicating its lower sensitivity than that of HMX. Furthermore, its energy content was found to be 20% more than that of TATB, thus LLM-105 has been regarded as a representative of insensitive and high-energy explosives similar to 1,1-diamino-2,2-dinitroethylene (FOX-7) [5,6]. Figure 1 shows the crystal structure of LLM-105. This material consists of monoclinic crystal with P21/n space symmetry group, as determined by single crystal X-ray diffraction (XRD) [7]. Averkiev et al. found through X-ray study that there are a large number of intramolecular and intermolecular hydrogen bonding in LLM-105 crystals [8]. Subsequently, He et al [9] confirmed the viewpoint of Averkiev through density functional theory (DFT) study. Their conclusion indicated that the hydrogen bonding plays an important role in the stability of the LLM-105. Existence of intramolecular hydrogen bonding makes LLM-105 a relatively stable planar molecule. Intramolecular and intermolecular hydrogen bonding result in a high crystal density of LLM-105 (1.9139 g cm^−3^) [10]. In the crystal, LLM-105 molecules are arranged and packed in zigzag layered structure via hydrogen bonding and π–π stacking. All these features could be invoked to explain the low mechanical sensitivity and high stability of LLM-105 [11,12]. 

From the practical perspective, the responses and mechanisms during storage and ageing as well as during performance should be concerned. Gump et al [13] investigated the isothermal equations of state (EOS) of LLM-105 at static high-pressure and temperature by synchrotron angle-dispersive XRD and diamond anvil cells for the first time. Thereafter, Manaa et al [14] reported dispersion-corrected DFT calculations of the unreacted equation of state of crystal LLM-105 under hydrostatic compression of up to 45 GPa. They estimated the heat of sublimation to be 32.4 kcal mol^−1^ and obtained solid heat of formation of LLM-105 to be −9.9 kcal mol^−1^ based on the G4 method. They found that with the increase in the pressure from 0 to 45 GPa, the band gap of the electron energy decreased from 1.3 to 0.6 eV. Then, Stavrou and Manaa [15] continued to measure the EOS by XRD and isobaric–isothermal atomistic molecular dynamics simulations, and results showed good agreement between the experimental and theoretically derived EOS. Recently, Wang et al [16] tried to determine the initial decay details of LLM-105 based on quantum chemistry calculations and molecular dynamics simulations. They presented four pathways for its decay, and found that the intramolecular H transfer from amino group to acyl O was a reversible process so that it could buffer against the external stimuli, which may partly be responsible for the low sensitivity of LLM-105.

There has been more than two decades since LLM-105 was first made. Numerous research reports have provided information about the characteristics and performances of LLM105. Yet, little is known about its thermal decomposition process, in particular the initial step in the reaction. Thus, this aroused our interest in the decomposition mechanism of LLM-105. In our previous research, we used the method proposed by Byrd and Rice [17] to predict the sublimation enthalpy of LLM-105 to be 24.7 kcal mol^−1^, while Manaa estimated this value to be 32.4 kcal mol^−1^ [14]. Significantly low sublimation enthalpy indicates that LLM-105 is prone to sublimation during heating. Temperature-dependent Fourier transform infrared (FTIR) [18] spectroscopy and thermal analysis experiments [19] proved that LLM-105 has a high tendency to sublimate. When the temperature reaches 260 °C, the existence of a large amount of gaseous LLM105 molecules is observed due to violent sublimation. It can be foreseen that increase in local temperature during storage and aging will result in significant sublimation, leading to the probable decomposition of LLM-105 molecule in the hot atmosphere.

Therefore, systematic exploration of the decomposition mechanism of gaseous unimolecular LLM-105 is highly desirable. Unimolecular behavior is the first step in the energy release reactions in both gas and condensed phases [20,21,22], and the unimolecular decomposition that triggers the energetic materials can provide key information about properties and decomposition details of these materials [23]. Furthermore, the revealed mechanism can provide a hand to the design of new energetic materials [23,24]. Therefore, unimolecular results represent a reasonable approximation to the primary, initial behavior for the decomposition of energetic molecules in general condensed phase materials [25]. 

In this study, the initial mechanism for unimolecular decomposition of LLM-105 was investigated and four possible initial reaction channels were proposed. The geometries, frequencies, energies, and thermodynamic parameters of the species on each path were obtained via quantum chemical computations. Intrinsic reaction coordinates (IRC) and energy barriers were calculated to confirm the intermediates and energy profiles of these channels, respectively. In order to debate the possibility of actual occurrence, kinetics analysis was conducted according to the transition state theory (TST) to find the dependences of rate constants on temperatures for these channels. Finally, the ab initio molecular dynamics simulation was used to verify the initial steps of unimolecular decomposition at different temperatures. 

## 2. Computational Methods

All quantum chemical computations were executed by using the Gaussian 09 software package [26]. Geometric optimization of critical points (minimum, transition state, intermediates, and products) was carried out at the M062X/6-311+G (d, p) level of theory [27]. Frequencies, zero-point energies, and thermal corrections to enthalpy and Gibbs free energy were calculated at the same level of theory. All the reactants, intermediates, and products have no imaginary frequencies, and every transition state should have only one imaginary frequency. Their single point electronic energies were calculated at the B2PLYPD3/aug-cc-pVTZ level of theory [28]. Calculation of the minimum energy paths was carried out by using the intrinsic reaction coordinate (IRC) algorithm. No symmetry restrictions were applied for all the calculations. Electron localization function (ELF) analysis and their color-filled maps were obtained by using the Multiwfn program [29]. 

In order to clarify the kinetics of initial decomposition channels, the rate constants of unimolecular reactions in this study were computed in accordance with the canonical TST [30,31] as following formula:

(1) $$k=\kappa \frac{k_{B}T}{h}\exp\left(-\frac{\Delta G^{‡}}{RT}\right)$$

where *κ* is a proportionality constant (referred to as the transmission coefficient), *k*_B_ is the Boltzmann constant, *h* is the Planck constant, *R* is the gas constant, *T* is thermodynamic temperature, and Δ*G*^‡^ is a free energy of activation. All rate constants were calculated in the temperature range of 300–600 K with a step size of 50 K and then approximated by using the Arrhenius equation as follows:

*k* = *A*exp(−*E*_a_/*RT*) (2)

where *A* is pre-exponential factor and *E*_a_ is activation energy.

Further, gas phase dynamics simulations were performed in the generalized gradient corrected approximation (GGA) using the Perdew-Burke-Ernzerh (PBE) exchange correlation functional [32] as implemented in CASTEP code [33]. The constant-temperature constant-volume (NVT) ensemble was used to simulate dynamics at fixed volume using a thermostat to maintain a constant temperature. The gamma point was selected as k-point that was used to integrate the wavefunction in reciprocal space. A plane wave basis with a kinetic energy cutoff of 700 eV was selected by convergence test. The electronic wavefunctions were obtained by using a Pulay density-mixing minimization scheme for the self-consistent field calculation [34], while the structures relaxation was implemented by the Broyden, Fletcher, Goldfarb, and Shanno (BFGS) method [35]. In the simulations, an optimized LLM-105 molecule was place in a cubic vacuum box with dimension of 20 × 20 × 20 Å^3^. The ab initio molecular dynamics computations were carried out at different temperatures to observe the initial decomposition reactions. Each simulation lasted for 2 ps with a time step of 0.5 fs. 

## 3. Results and Discussion

### 3.1. Initial Decomposition Channels

**Figure 2:**
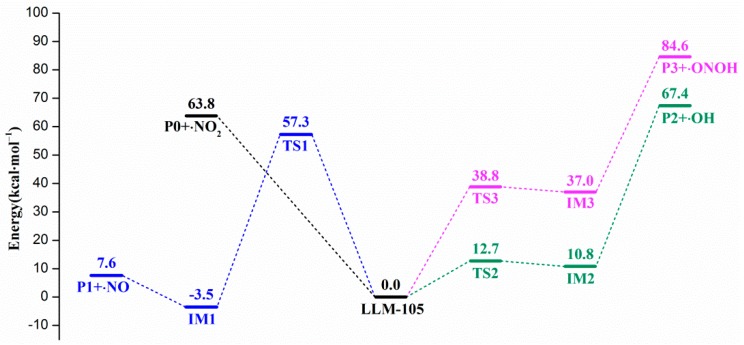
Calculated minima and transition states for the possible initial dissociation channels of LLM-105 on the ground state potential energy surface.

**Figure 3:**
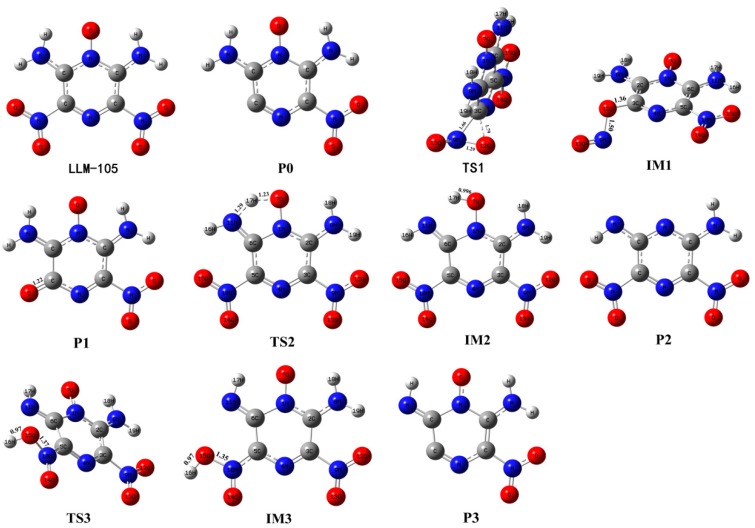
Calculated geometries for stationary points shown in Figure 2. The C, H, O, and N atoms are represented in gray, white, red, and blue, respectively.

There are four reaction channels as candidates for the initial pathways in the gas phase decomposition of LLM-105 according to our calculated results. That is, the C–NO_2_ bond homolysis; the NO elimination after the nitro–nitrite rearrangement [36] the H transfer from amino to acyl O or to nitro O followed by OH or HONO elimination, respectively [37,38,39]. The C–NO_2_ homolysis was modeled by moving the NO_2_ group away along the C–NO_2_ bond direction. To simulate the nitro–nitrite rearrangement, the C–NO_2_ bond was extended and NO_2_ was slightly rotated. After the relaxation, one of the O atoms of the NO_2_ group binding on the C atom of the C–NO_2_ bond, combined with the corresponding N–O bond cleavage to form the CONO isomer. The two types of H transfer were modeled by transferring H atom from amino to acyl O and to the O atom in ortho-nitro group, respectively. All the energy profiles of the stationary points are shown in Figure 2. The geometries of LLM-105, transition state species, intermediates, and corresponding decomposition products are given in Figure 3, with the relevant geometric parameters labeled when needed.

Guirguis and co-workers [40] pointed out that the thermal decomposition of nitro-containing compounds usually begins with the dissociation of C–NO_2_ or N–NO_2_ bond. For the compounds such as nitroaliphatics, nitramines, and nitropyrazines, the X–NO_2_ bond is usually the weakest bond in the molecule,^1^ where X can be C, N or O. In this study, the C–NO_2_ bond was gradually elongated for the flexible scanning; however, the transition state for this bond dissociation reaction was not obtained. Thus, the energy barrier of this reaction was obtained by calculating the bond dissociation energy (BDE) of C–NO_2_ bond. Figure 2 shows that the BDE of C–NO_2_ in LLM-105 is 63.8 kcal mol^−1^, which is consistent with the BDE ranges (61–70 kcal mol^−1^) [1,41] for many nitro compounds, e.g., nitrobenzene (71 kcal mol^−1^) [1,42], TNB (64.0 kcal mol^−1^), TATB (69.4 kcal mol^−1^) [43], and so on. The fission of C–NO_2_ yields a nitropyrazine radical (P0) and NO_2_, and then the P0 further undergoes decomposition to produce final products.

**Figure 4:**
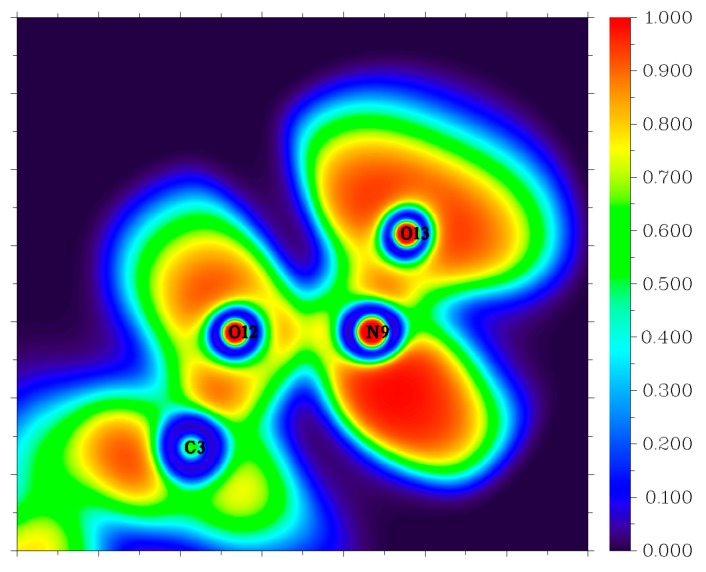
Color-filled electron localization function (ELF) map of C–N–O three-center ring in TS1.

**Figure 5:**
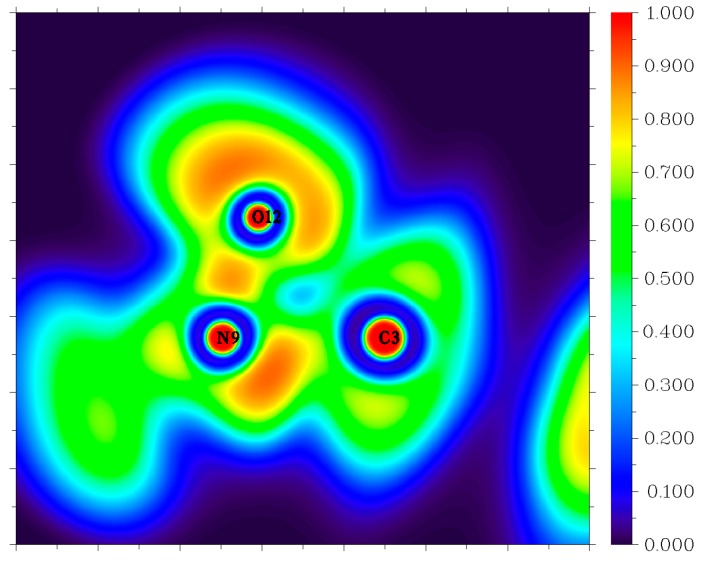
Color-filled electron localization function (ELF) map of C–O–N–O in IM1.

Though the dissociation of C–NO_2_ is recommended as the dominant decomposition path for the nitro-containing aromatic compounds, there is evidence that this is not always the case. A typical fact is that the thermal decomposition of nitrobenzene in the gas phase is insensitive to the presence of excess NO_2_ [44]. The nitro–nitrite rearrangement on an aromatic backbone could be very important in the initial steps relative to C–NO_2_ homolysis [1]. In the present study, the LLM-105 molecule could form a transition state (TS1) with a barrier of 57.3 kcal mol^−1^. The TS1 has a traditional nitro–nitrite isomerization geometry with a C–N–O three-member ring structure, which is almost perpendicular to the pyrazine backbone plane. In this ring structure, the C3–O12 bond length is 1.79 Å. The C3–N9 and N9–O12 bond lengths are 1.66 and 1.29 Å respectively, which are longer than the corresponding values in LLM-105 (1.47 and 1.20 Å, respectively). Electron localization function (ELF) [45,46] analysis (see Figure 4) of TS1 shows strong electronic localization between C3 and N9, indicating a strong covalent bond between these two atoms [47]. Moreover, relatively small ELF value between C3 and O12 indicates that the covalent bonding between them has not been fully formed yet. This actually meets the feature of the transitional state of chemical reaction. After crossing the transition state, TS1 transforms to a stable intermediate (IM1) accompanying an exothermic value of 60.8 kcal mol^−1^. In IM1, C3–O12 bond length shortens to 1.36 Å, the ELF map indicates a firm covalent bonding between these two atoms (see Figure 5). Moreover, C3–N9 bond completely ruptures, and N9–O12 bond elongates to 1.50 Å; however, still remains as strong covalent interaction (see Figure 5). Then, IM1 undergoes endothermic process to release NO and yields P1 radical. The BDE of this C–NO bond is only 11.1 kcal mol^−1^, thus this NO loss occurs quite easily.

**Figure 6:**
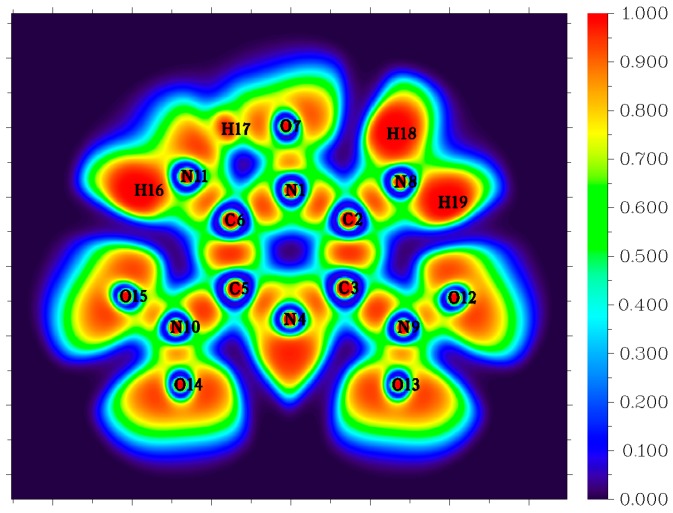
Color-filled electron localization function (ELF) map of TS2.

The third channel for initial decomposition of LLM-105 is a hydrogen transfer from the amine to its neighbor acyl O with subsequent OH elimination, as shown in green in Figure 2. First, H17 approaches O7 to form a five-number ring-like transition state TS2 with a barrier of only 12.7 kcal mol^−1^ relative to LLM-105. ELF map of TS2 (Figure 6) shows darker orange/red coloration between N11–H17 and O7–H17, indicating strong covalent interaction in these two bonds. As H17 goes closer to O7, the O7–H17 covalent bonding is enhanced accompanied by the rupturing of N11–H17 bond, eventually forming the intermediate IM2 with a slight exothermic value of 1.9 kcal mol^−1^ relative to TS2. Then the H17 expects to migrate back from IM2 to amino group attributed to the quite low reverse barrier. Another possibility is that IM2 cracks to yield P2 and hydroxyl radicals in the case of continued heat absorption of 56.6 kcal mol^−1^. This type of H shift process seems to occur easily on grounds of its quite low barrier compared to C–NO_2_ bond homolysis. The result is consistent with the findings of Wang et al., indicating that H transfer dominates the initial fission of LLM-105 at relatively low temperatures due to its low energy barrier [16].

**Figure 7:**
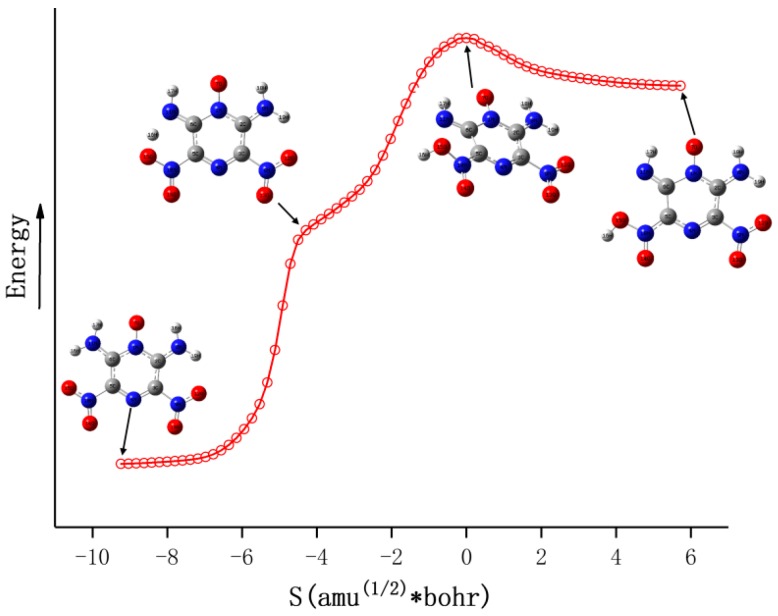
Intrinsic reaction coordinate (IRC) profile of the pathway from LLM-105 to IM3.

Given the fact that there is an adjacent nitro group to each amino group in the LLM-105 molecule, the H transfer from amino group to the O on ortho-nitro is also a possible initial decomposition pathway. Figure 2 exhibits the plot of the energy profile of this pathway in purple. According to our calculations, H transfers from the amino to the oxygen on the adjacent nitro group to form a transition state TS3 with the barrier of 38.8 kcal mol^–1^. In this transition state, H–O bond with a bond length of 0.97 Å was completely formed. The H turned to one side of the nitro group with –ONOH group twisted and bent out of the plane. Evidently, this geometry was different from that of TS2, in which H moved to the position between the N and O and formed a five-member ring that was almost coplanar with the pyrazine backbone. This unusual geometry (TS3) was confirmed by the IRC calculation (Figure 7) which clearly shows that when H shifted to the point between N and O, its energy did not reach the maximum but a shoulder along the reaction coordinate. Until H bonded with O and turned to the position out of the plane, energy became the maximum along the IRC, and this is the saddle point called TS3. Accompanying a slight exotherm, H rotated to the same plane of nitro group, and the entire molecule returned to a nearly planar structure, forming intermediate IM3. Of course, this intermediate is unstable, it can easily back to the TS3 and then to LLM-105 ground state molecule, or can release HONO radical by rupturing the C–NO_2_H bond with absorption of 47.6 kcal mol^−1^ of energy.

### 3.2. Kinetics Analysis

**Figure 8:**
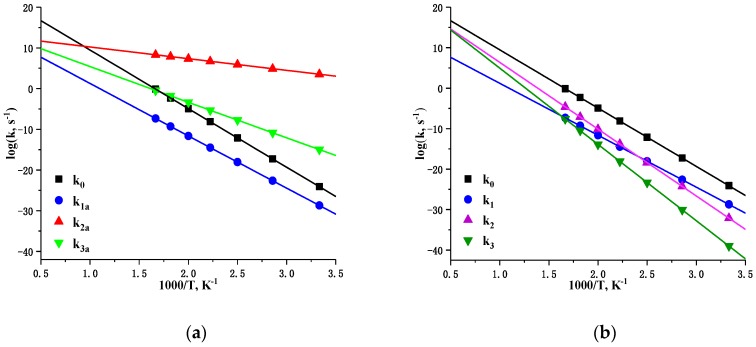
The logarithmic dependences of rate constants (logk) on temperatures (1000/T) for initial decomposition channels of LLM-105 molecule. Subfigure (**a**) shows the first steps of four channels and (**b**) shows the whole reactions of four channels.

The rate constants for initial reactions were calculated in the temperature range of 300–600 K with a step size of 50 K, and then the rate constants were approximated and extrapolated by using the Arrhenius equation. The logarithmic dependences of rate constants (log*k*) on temperatures (1000/*T*) were fitted and shown in Figure 8. These linear relations provided activation energies (*E*_a_) and pre-exponential factors (*A*) for reactions which are listed in Table 1. Figure 8(a) exhibits that the reaction of H transfer to acyl O is the fastest at 1200 K, and its rate constant (*k*_2a_) can reach 10^4^ at ambient temperature. However, it grows slowly with increasing temperature as this reaction has the lowest preexponential factor A. The *k*_2a_ gets exceeded by *k*_0_ beyond 1200 K, as well as by *k*_1a_ and *k*_3a_ at higher temperatures. The entire OH elimination reaction rate is slow at low temperatures due to the activation energy being up to 75.5 kcal mol^−1^. In high temperature regions, the rate of this dissociation channel is greatly accelerated, but still unable to compete with C–NO_2_ bond dissociation (see Figure 8(b)). With respect to the HONO elimination channel, the first step of H transfer seems faster than the direct C–NO_2_ bond homolysis in the low temperature range, soon the former gets outstripped by the latter at 580 K. Yet the entire HONO elimination reaction is the slowest below 600 K. Above this temperature, rate constant rapidly increases with increasing temperature. Even so, it is still below the rates of NO_2_ partition and OH elimination unless at extremely high temperatures. As previously shown in Figure 2 that the NO elimination reaction (LLM-105 → P1 + •NO) should overcome the same barrier as to nitro–nitrite rearrangement, thus their rate constants are the same according to our calculations. Although NO elimination reaction has the lowest activation energy in the case of LLM-105 decomposition, it also has the minimum pre-exponential factor. However, Figure 8 shows that nitro–nitrite rearrangement is relatively slow, indicating that this rearrangement and following elimination reaction only contribute at low temperatures. Thus, when the temperature is above 400 and 600 K, this reaction rate is exceeded by that of OH elimination and HONO elimination, respectively. Furthermore, rate constant of C–NO_2_ homolysis is unfavorable at low temperatures; however, it increases rapidly and ultimately surpasses two types of hydrogen transfer reactions at the temperatures above 1200 K. Therefore, C–NO_2_ homolysis dominates in the initial decomposition of LLM-105 at high temperatures owing to its highest pre-exponential factor (log*A* = 23.9). The channels of OH elimination and HONO elimination have larger activation energies and high pre-exponential factors that are close to those of the C–NO_2_ bond dissociation, indicating that they will contribute more to the initial decomposition of LLM-105 under very high temperatures. 

Owing to low barrier and reversibility of H transfer from amino group to the acyl O, such H transfer can buffer against thermal stimulation during moderate heating. Assuming that the temperature continues to increase, once LLM-105 molecule accumulates sufficient energy to overcome the barrier of 38.8 kcal mol^−1^ H atom can migrate to the O of nitro group. However, this type of H transfer is also reversible, as well it has high rate, thus it can buffer against thermal stimulation at this stage. Above 580 K, C–NO_2_ bond dissociation proceeds faster than the transfer of H to the nitro group. Combined thermal analysis results indicated that mass loss of LLM-105 and heat release started at about 580 K and decomposition temperature peak was 615 K (342 °C). We would suggest that it is the occurrence and rapid progressing of C–NO_2_ dissociation that led to the final thermal decomposition. This analysis indicates that NO_2_ loss reaction is maybe the real initial channel, at least a decisive step, in the thermal decomposition of LLM-105. At higher temperatures, sufficient energy is present to facilitate the OH or HONO elimination after corresponding H transfer, respectively. These elimination reactions provide the rate constants that can approximate the C–NO_2_ dissociation, signifying that these two channels contribute to initial decomposition of LLM-105 at high temperatures. 

The obtained results clearly indicate that LLM-105 is more thermally stable than most known high-energy explosives and is nearly identical to TATB [10]. This is attributed to two types of reversible H transfer that can buffer against thermal accumulation during heating. In particular, the H transfer between amino and nitro, which also occurs frequently in TATB, could be invoked to explain the fact that thermal stability of LLM-105 is almost equal to that of TATB. 

### 3.3. Molecular Dynamics Simulations

**Figure 9:**
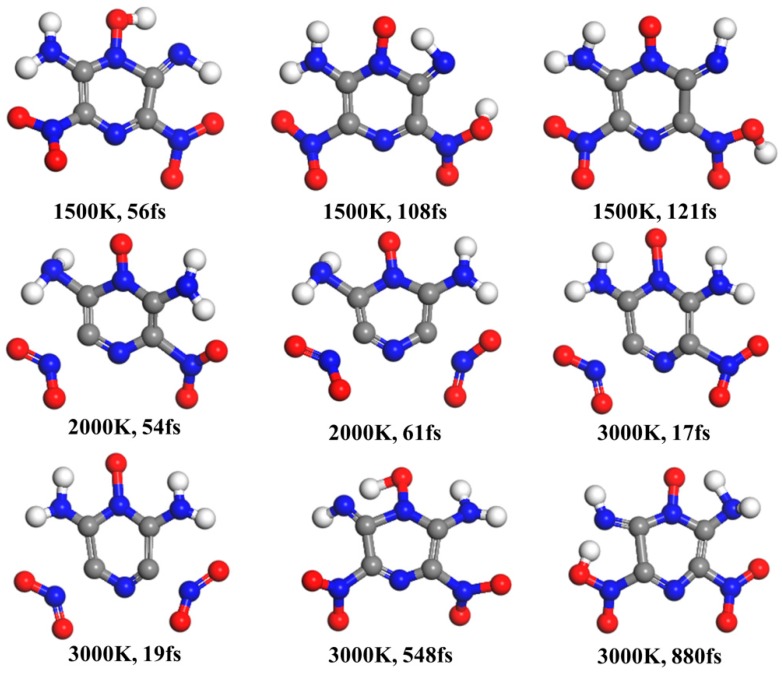
Snapshots of initial decomposition step of LLM-105 molecule in dynamics simulations.

Ab-initio molecular dynamics simulations were carried out to observe the thermal decomposition behavior of LLM-105 molecule in the gas phase. We selected 1500, 2000, and 3000 K for constant-temperature heating on account of the fact they are appropriate temperatures for simulations to search primary steps within the limited time scale. Figure 9 illustrates that at 1500 K, first the H migrates to acyl O at 56 fs and then it returns back to the amino group at 105 fs. Further, H transfer occurs between amino and nitro groups. At 108 fs, the transition state geometry is obtained as calculated above (see Figure 3), and the intermediate IM3 appears at 121 fs. Soon, the H returns back to amino group at 164 fs. Further, these two types of H transfer occur several times, and the former is more frequent than the latter. However, the C–NO_2_ bond dissociation is not observed within 2 ps, explicating that it may take longer time to accumulate enough energy to make C–NO_2_ bond rupture at this temperature. At 2000 K, the primary reaction is the C–NO_2_ homolysis at 54 fs, and then another C–NO_2_ bond ruptures at 61 fs. Two types of H transfer occur several times; however, their lifetime is very short (< 20 fs) [48], thus we could not determine whether it really happened or it was just instantaneous fluctuations. At the constant temperature of 3000 K, the two C–NO_2_ bonds dissociations occurred at 17 and 19 fs, respectively. The reversible H transfer between amino group and acyl O was captured from 548 to 642 fs. This type of H transfer and C–NO_2_ homolysis appeared several times again afterwards. We found that the H migrated to the nitro O at 880 fs, and was released back to amino group soon. The dynamics simulations in this study did not explain the nitro–nitrite rearrangement, mainly attributed to its higher barrier and lower rate constant than other reactions.

### 3.4. Discussion

The X–NO_2_ bond scission is a key reaction or even a decisive step in the initiation of thermal decomposition of many nitro-containing explosives, and thus can be correlated with the sensitivity of these explosives [49,50,51]. In the case of LLM-105, C–NO_2_ homolysis is the dominant channel of the initial pathways for dissociation processes based on its moderate reaction energy barrier and high rate constants over large temperature ranges. Although nitro–nitrite rearrangement has lower barrier compared to C–NO_2_ bond scission and easily releases NO radical immediately, its pre-exponential factor is much smaller than that of other pathways that causes lower rate constants especially at high temperatures. Therefore, it was difficult to observe this reaction based on dynamics simulations. The barrier corresponding to the migration of H to acyl O is so low that it can occur even at ambient temperature. However, this type of H transfer has been proven to be a reversible process, which can buffer the external stimuli [16]. Moreover, further dissociation of N–OH bond requires an extra endothermic value of 56.6 kcal mol^–1^, indicating that OH elimination is hard to actually happen. This conclusion was also affirmed by kinetics analysis, which indicated that the reaction rate of this pathway was much lower than that of C–NO_2_ homolysis at all temperatures. As a matter of fact, dynamics simulations in this study indicated that this type of H transfer occurred frequently; however, the subsequent N–OH dissociation was not observed. Another type of intramolecular H transfer between amino and nitro groups has a barrier much higher than the former, but visibly lower than the C–NO_2_ homolysis or the nitro–nitrite rearrangement. Similar to the former H transfer case, the reverse process of this type of H transfer requires almost no energy consumption. Thus, one can speculate that this type of H transfer is reversible too, thereby buffering against the enhanced thermal stimulation from increased temperature. Furthermore, kinetics analysis and thermal analysis data revealed that LLM-105 exhibited good thermal stability until this H transfer rate was exceeded by the rate of C–NO_2_ homolysis at above 580 K, providing evidence for our speculation. Calculation showed that additional 47.6 kcal mol^–1^ of energy was required to release HONO radical after H transfer, causing the entire HONO elimination pathway to have lower rate compared to NO_2_ loss or OH elimination within the reaction temperature range. As expected, we detected the formation of HONO isomer a few times based on the dynamic simulations; however, subsequent HONO elimination could not yet be observed. All HONO isomers eventually returned back to the LLM-105 molecular structure, demonstrating the reversibility of this type of H transfer.

This study is mainly about the mechanism for gas phase unimolecular decomposition of LLM-105; however, in the condensed phase there may be differences. In the crystal, LLM-105 molecules are packed by *π–π* stacking to form a zigzag-like layered structure. The presence of intramolecular and intermolecular hydrogen bonding complicates the stresses of hydrogens and nitro groups. Intermolecular hydrogen transfer probably becomes frequent, while nitro–nitrite rearrangement is expected to be more difficult to occur under the influence of hydrogen bonds and steric hindrance. Moreover, intermolecular hydrogen transfer may couple with the NO_2_ partition. Such coupling interactions between the initial decomposition reactions are likely to be the key steps in the decomposition of LLM-105 crystal, thereby affecting their sensitivities and explosive performances. The corresponding investigations will be implemented in our next program. 

## 4. Conclusions

The initial channels of thermal decomposition mechanism of 2,6-diamino-3,5-dinitropyrazine-1-oxide (LLM-105) molecule were investigated. The results of quantum chemical calculations revealed four steps involved in the reaction pathway, including the C–NO_2_ bond homolysis, nitro–nitrite rearrangement followed by NO elimination, and H transfer from amino to acyl O and to nitro O with the subsequent OH or HONO elimination, respectively. The results indicated that when the temperature was below 580 K, the H transfer from amino group to acyl O exhibited the lowest barrier and the highest rate, thus it could happen frequently even at ambient temperatures. Another type of H transfer between amino and nitro group exhibited barrier and rate second only to the former. These two types of H transfer seemed to dominate the initial decomposition at this temperature stage. However, the subsequent OH or HONO elimination reactions were difficult to occur for their additional energy consumption. Moreover, they were reversible reactions due to their extremely low reverse barriers, connoting that they could buffer against external thermal stimulation to different degrees, which were mainly responsible for the thermal insensitivity of LLM-105. Above 580 K, C–NO_2_ homolysis rate surpassed the latter type of hydrogen transfer. Moreover, at such temperatures, it was easy to accumulate enough energy to rupture the C–NO_2_ bond. Therefore, the C–NO_2_ bond dissociation reaction occurred more often, leading to the rapid decomposition of LLM-105. This is consistent with the results of many thermal analysis experiments that rapid exothermic decomposition of LLM105 occurs at about 300 °C (around 580 K) and decomposition temperature peak is 342 °C (615 K). At high temperatures, e.g., above 2000 K, the rate of C–NO_2_ homolysis was significantly higher than that of H transfers. Even though the OH or HONO elimination reaction rates were significantly improved, they still could not compete with C–NO_2_ homolysis from a kinetic point of view. 

In summary, we tentatively propose that the C–NO_2_ bond dissociation dominates the initial thermal decomposition of LLM-105 molecule. Two types of reversible H transfer play a role in buffering against thermal stimulation to maintain thermal stability of LLM-105 molecule before the decomposition. The corresponding OH or HONO elimination following H transfer may contribute to the primary decomposition reaction at very high temperatures. The rates of the nitro–nitrite rearrangement and the subsequent NO elimination are always lower than those of other reactions, thus it could not be the initial decomposition channel. Noteworthy, our conclusions are gained and speculated in the case of unimolecular gas phase decomposition. These results may be anfractuous with regard to the thermal decomposition mechanism of condensed phase LLM-105. Our findings are expected to give insights into the thermal stability and decomposition mechanism of LLM-105.

## Figures and Tables

**Table 1:** Preexponential factors *A* and activation energies *E*_a_ for four candidates of initial thermal decomposition channels of LLM-105 molecule.

Reaction		Log(*A*, s^−1^)	*E*_a_, kcal mol^−1^
**LLM-105** **→** **P_0_+** **•** **NO_2_**	(k_0_)	23.9	65.8
**LLM-105** **→IM1**	(k_1a_)	14.0	58.7
**LLM-105** **→P_1_+** **•** **NO**	(k_1_)	14.0	58.7
**LLM-105** **→IM2**	(k_2a_)	13.1	13.1
**LLM-105** **→P_2_+** **•** **OH**	(k_2_)	22.9	75.5
**LLM-105** **→IM3**	(k_3a_)	14.2	40.0
**LLM-105** **→P_3_+** **•ON** **OH**	(k_3_)	23.8	86.2
